# Intravascular Ketamine Increases Theta-Burst but Not High Frequency Tetanus Induced LTP at CA3-CA1 Synapses Within Three Hours and Devoid of an Increase in Spine Density

**DOI:** 10.3389/fnsyn.2018.00008

**Published:** 2018-05-30

**Authors:** Allie J. Widman, Amy E. Stewart, Elise M. Erb, Elizabeth Gardner, Lori L. McMahon

**Affiliations:** ^1^Department of Cell, Developmental and Integrative Biology, University of Alabama at Birmingham, Birmingham, AL, United States; ^2^Department of Criminal Justice, University of Alabama at Birmingham, Birmingham, AL, United States

**Keywords:** hippocampus, anti-depressants, ketamine, synaptic plasticity, spine density

## Abstract

In the past 20 years, ketamine has become a promising treatment for Major Depressive Disorder (MDD) due to its rapid and sustain antidepressant effects in patients. A single ketamine treatment causes improvement in depressive symptoms within hours and can last weeks, long after it is eliminated. Previous studies have demonstrated increased synaptic plasticity at CA3-CA1 synapses in hippocampus (HPC) 24 h post ketamine treatment suggesting increased activity-dependent hippocampal function may underlie the antidepressant effects of ketamine. If true, these changes should also occur within hours of treatment, a time when symptoms are first alleviated in patients. To determine if augmented synaptic plasticity is observed at an earlier time point, we measured theta-burst and high frequency tetanus induced long-term potentiation (LTP) at CA3-CA1 synapses 3 h following intravenous (IV) ketamine administration. Additionally, we measured basal hippocampal function and spine density to investigate whether connectivity was increased with ketamine treatment. We report that theta-burst but not high frequency tetanus induced LTP is significantly increased 3 h after *in vivo* ketamine with no changes in basal synaptic function or morphology. Our finding supports increased activity-dependent hippocampal function underlying the antidepressant effects of ketamine as it occurs at a time point that correlates with initial improvements of depressive symptoms in patients.

## Introduction

Understanding how ketamine, a psychoactive drug of abuse, causes rapid antidepressant effects has been a priority, as it has the ability to alleviate depressive symptoms within 1–3 h of intravenous (IV) administration (Berman et al., 2000; Zarate et al., 2006; Fond et al., 2014) and improve cognition within days of treatment (Lara et al., 2013). With Major Depressive Disorder (MDD) having a lifetime prevalence of 17% (Kessler et al., 2005) and first-line antidepressants taking weeks or months to improve symptoms (Trivedi et al., 2006), this rapid action of ketamine is promising for many. However, widespread use of ketamine may be limited due to psychotomimetic side effects, prompting studies to determine the mechanism of action so alternatives can be developed. Fortunately, the rapid antidepressant-like behavioral effect of ketamine is mimicked in rodents (Garcia et al., 2008, 2009; Maeng et al., 2008; Li et al., 2010, 2011; Autry et al., 2011; Burgdorf et al., 2013), providing an experimental model within which mechanisms contributing to the anti-depressant and cognitive enhancing effects can be determined.

Most preclinical studies have assessed the impact of ketamine on glutamate transmission and spine density at 24 h post-injection. Ketamine increases spine density 24 h post treatment in prefrontal cortex (PFC; Li et al., 2010, 2011; Burgdorf et al., 2013) and area CA1 (Liu et al., 2016), suggesting increased connectivity in these brain regions, which may counteract the neuronal atrophy implicated in the pathophysiology of MDD (Neumeister et al., 2005). Functionally, AMPA receptor (AMPAR)-mediated transmission is augmented in PFC (Li et al., 2010, 2011), and studies in hippocampus (HPC) report enhanced long-term potentiation (LTP) in area CA1 24 h after ketamine administration, indicating increased circuit function (Burgdorf et al., 2013; Graef et al., 2015). Yet, these studies have not assessed how the circuit has changed during the time frame in which humans initially experience improved mood.

In addition to the lack of information on how ketamine impacts circuit function during the first few hours following administration, a second limitation of many preclinical studies is the intraperitoneal (IP) rather than the IV route of ketamine administration used in humans, which enables ketamine to reach brain within 1 min (Cohen et al., 1973). Our previous *in vitro* study demonstrates bath application of 1 μM ketamine increases synaptically driven CA1 pyramidal excitability (Widman and McMahon, 2018). In support, another study showed ketamine (1 μM) bath application increased CA1 somatic excitatory postsynaptic potentials (EPSPs) compared to the dendritic EPSP (Izumi and Zorumski, 2014), suggesting an enhanced pyramidal cell excitability in the presence of ketamine. Additional studies demonstrate AMPAR-mediated transmission is enhanced within 1 h after bath application of ketamine (20 μM) in HPC (Autry et al., 2011; Nosyreva et al., 2013; Zhang et al., 2016), although these studies used ketamine concentrations at least two times higher than what is thought to reach brain in humans (Hartvig et al., 1995). These findings indicate ketamine likely augments function as soon as it reaches the brain, which will be within minutes following an IV injection. Interestingly, ketamine increases release of BDNF, and the antidepressant-like effects of ketamine rely on BDNF (Lepack et al., 2014). However, it is unknown whether the increased activity of CA1 pyramidal cells and BDNF release with ketamine may enhance BDNF-dependent plasticity within hours of treatment.

If increased circuit function in HPC is involved in the antidepressant efficacy of ketamine, these changes should be occurring as soon as the antidepressant behavioral effect is observed. Therefore, we examined whether ketamine increases hippocampal circuit function at 3 h post injection. In addition, we determined whether an increase in dendritic spine density might also be observed in area CA1 and PFC at this early time point. Finally, we used gas chromatography/mass spectrometry (GC/MS) to determine the time frame at which ketamine remains in brain to correlate with possible changes in synaptic function. Importantly, we administered ketamine IV to mimic the route of administration inpatients. We found that 3 h post treatment, ketamine was undetectable in brain, yet we observed increased LTP magnitude induced using theta burst stimulation (TBS) but not high frequency stimulation (HFS), in the absence of changes in basal synaptic transmission and dendritic spine density.

## Materials and Methods

All experimental procedures were approved by the University of Alabama at Birmingham’s Institutional Animal Care and Use Committee and were performed in accordance with National Institutes of Health experimental guidelines.

### Animals and Injections

Male Sprague-Dawley rats (2–4 months old; Charles River Laboratories) housed in a 12 h light/dark cycle with free access to food and water were used throughout the study. For IV administration, ketamine (100 mg/ml) was diluted to 20 mg/ml with sterile saline, and rats were given a 10 mg/kg ketamine dose or equal volume of saline directly into the lateral tail vein. During the injection, animals were briefly restrained using a decapicone (Braintree Scientific, Braintree, MA, USA).

### Gas Chromatography—Mass Spectrometry (GC/MS)

Rats were rapidly decapitated at 0 min, 30 min and 3 h following IV ketamine administration and HPC, PFC and cerebellum were collected. Sample preparation and analyte extraction techniques were adapted from a method for extracting ketamine provided by DPX Technologies (Columbia, SC, USA). Samples were weighed and added to a 2 ml snap vial with 10–15 metal beads (2.4 mm). Then, 500 μL water and 50 μL of the internal standard, ketamine-d4 (100 mg/L), were added. Tubes were vortexed until the tissue sample was fully homogenized and centrifuged at 12.5 × 1000 rpm for 15 min in an Eppendorf Minispin centrifuge. The supernatant was transferred to a new tube containing 1 mL of acetonitrile in order to precipitate proteins. Tubes were vortexed for a minute and centrifuged at the same speed for another 15 min. The aqueous layer was added to a test tube containing 2 mL of sodium acetate buffer (0.1 M, pH 5) to begin the extraction procedure. Solid-phase extraction (SPE) dispersive pipette tips (5 ml, 5S-5TF25-02-030-050-5B DPX Technologies) were used to perform the extraction of ketamine and norketamine (NK). The tips were conditioned by aspirating and dispensing 3 mL of methanol followed by 3 mL of water. Then the liquid sample was aspirated for 15 s and dispensed from the tips; this step was repeated four times. Next, 2 mL of sodium acetate buffer were aspirated and dispensed one time, followed by 2 mL of methanol. Lastly, 3 mL of a 3% ammonium hydroxide solution in acetonitrile (pH 10) was aspirated and dispensed into a clean test tube and evaporated to dryness under nitrogen in an Organomation Associate’s N-EVAPTM112. The residue was dissolved in 100 μL of hexane and transferred to 200 μL microvials for GC/MS analysis with an Agilent 6890N Network GC system/5975 inert Mass Selective Detector with selected ion monitoring. The ions used for quantitation for ketamine, ketamine-d4 and NK were 180, 184 and 166 respectively. Qualifier ions used for ketamine were 138 and 152 and for NK were 131 and 195. A calibration curve for quantifying the ketamine and NK was created by preparing solutions of known concentrations of ketamine and NK. These solutions went through the extraction process in order to account for any loss of the analytes occurring during the extraction process. Calibration curve standards were prepared at 1, 3, 5, 10 and 15 ng/μL each week from ketamine, NK and d_4_Ketamine standards (1 mg/mL) purchased from Cerilliant and a calibration curve was prepared based on the results. The line of best-fit equation was generated in Microsoft Excel and used to calculate concentrations in samples. Calibration curves were linear for all of the analytes with *r^2^* values ranging from 0.98 to 0.99, and the limit of quantitation for both ketamine and NK was 1 ng/μL.

### Hippocampal Slice Preparation

Three hours post ketamine or saline injection, rats were decapitated following isoflurane anesthesia. Hippocampal slices were prepared as previously described (Stewart et al., 2017; Smith and Mcmahon, 2018; Widman and McMahon, 2018). Briefly, brains were rapidly removed and placed in cold, high-sucrose, low-Na^+^ artificial cerebrospinal fluid (aCSF) containing (in mM): 85 NaCl, 2.5 KCl, 4 MgCl_2_, 0.5 CaCl_2_, 1.25 NaH_2_PO_4_, 25 NaHCO_3_, 25 glucose and 75 sucrose. Coronal slices (400 μM) were cut using a vibratome (Leica VT1000P) from dorsal HPC in cold, high-sucrose, low-Na^+^ aCSF equilibrated with 95% O_2_ and 5% CO_2_. Slices were allowed to recover for 1 h at room temperature in standard aCSF containing (in mM): 119 NaCl, 2.5 KCl, 1.3 MgCl_2_, 2.5 CaCl_2_, 1.0 NaH_2_PO_4_, 26 NaHCO_3_ and 11 glucose equilibrated with 95% O_2_ and 5% CO_2_.

### Electrophysiology

Slices were placed in a submersion chamber and continuously perfused (3–4 ml/min) with standard aCSF equilibrated with 95% O_2_ and 5% CO_2_ and maintained at 26–28°C. Extracellular field excitatory postsynaptic potentials (fEPSPs) at CA3-CA1 synapses were recorded by stimulating Schaffer collaterals with pairs of pulses (0.1 Hz, 100 μs duration at 50 ms interval) in stratum radiatum with a twisted nichrome wire bipolar electrode and recorded with a glass pipet filled with aCSF placed nearby in stratum radiatum. To generate stimulus-response curves, the stimulus intensity was increased by 10 μA intervals, from a threshold stimulus of 20 μA, until a maximum fEPSP was achieved. At least five fEPSPs were recorded at each stimulus strength and averaged to obtain a single value. Paired pulse ratio (PPR) was measured during baseline recording using pairs of pulses delivered at a 50 ms interstimulus interval. PPR was calculated by dividing the slope of the second fEPSP by the first. After a 20 min stable baseline of recording, LTP was induced using either TBS or HFS. TBS consisted of 10 bursts of five pulses at 100 Hz. The intraburst interval was 200 ms and the train was repeated four times with a 20 s interval between trains. HFS consisted of 2–100 Hz trains at 1 s duration with a 20 s interval between trains. Following TBS or HFS, stimulation returned to baseline frequency (0.1 Hz) and the fEPSPs were recorded for at least 40 min. The last burst in the TBS and the second train of HFS were used to measure steady state depolarization.

### Golgi Staining

For morphology studies, brains were processed using the FD GolgiStain Kit (FD NeuroTechnologies) according to the manufacturer’s protocol. Rats were anesthetized with isoflurane and rapidly decapitated 3 h after either IV ketamine or saline injection. The brain was removed and one hemisphere was submerged in Solutions A and B for 14 days and then transferred to Solution C for 72 h. Next, brains were embedded in 3% agar and cut into 150 μm coronal sections using a vibratome. Slices were mounted onto 3% gelatin coated slides, and were rinsed with ddH_2_O before being placed into Solutions D and E for development. Then, sections were dehydrated with increasing ethanol concentrations, cleared in xylene, and coverslipped with Eukitt mounting medium.

Microscopy was performed on a MicroBrightField system (MBF Bioscience). Stereo Investigator software was used to take z-stack images of tertiary dendrites from CA1 pyramidal cells in HPC and secondary dendrites of layer V pyramidal cells in PFC at 100× magnification. Criteria for selecting dendrites included: 10–40 μm length with no branch points, dendrites could not span more than 20 μm in depth, and dendrites had to be traceable back to the cell body. For each animal, a total of 5–9 sections of dendrites that met criteria were imaged from HPC and PFC of each rat. Spine density (number of spines/10 μm section of dendrite) was analyzed using Neurolucida with the experimenter blind to the treatment group. The dendrite was traced manually and spines were traced using a point-and-click method within Neurolucida.

### Analysis and Statistics

For electrophysiology experiments, data were acquired using Clampex 10.3 (pClamp, Molecular Devices, Sunnyvale CA, USA) and analyzed offline in Clampfit 10.3. An unpaired two-sample *t*-test was used to test for statistical significance using Origin 9 (Origin Labs, Northampton, MA, USA) or Prism 7 (GraphPad, La Jolla, CA, USA); significance was set to *p* < 0.05. All datasets are represented at mean ± SEM and graphs were composed in Prism 7.

## Results

### Ketamine Reaches Hippocampus Within Seconds Following IV Administration

**Figure 1:**
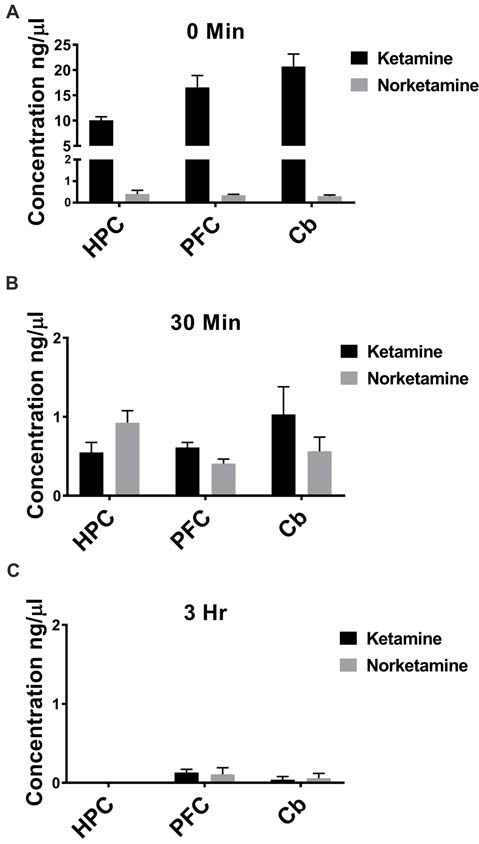
Ketamine is quickly eliminated from brain following intravenous (IV) injection. **(A)** Ketamine is detected in hippocampus (HPC), prefrontal cortex (PFC) and cerebellum (Cb) immediately following IV injection in the lateral tail vein. Low levels of norketamine (NK) are observed in each brain region at this timepoint (*n* = 6 animals). **(B)** Thirty minutes following IV injection, levels of ketamine are reduced and more NK is detected in each brain region (*n* = 5−6 animals). **(C)** By 3 h after IV injection, ketamine and NK are undetectable in HPC and a small amount of ketamine and NK is measured in PFC and Cb (*n* = 5−6 animals). All values are mean ± SEM.

IV administration is the fastest route possible to deliver drugs, leading to rapid distribution throughout the body. Because patients receive ketamine as an IV infusion, ketamine should reach brain within seconds and could potentially trigger immediate changes in brain circuits that lead to enhanced connectivity. To test whether ketamine reaches brain within minutes of an IV injection in rats and is rapidly cleared, we performed GC/MS. Specifically, we investigated the concentration of ketamine and its metabolite, NK, in PFC, HPC, and cerebellum. Immediately following an IV injection, ketamine is detected in PFC, HPC and cerebellum with little to no NK (Figure 1A, *n* = 6 animals). After 30 min, lower levels of ketamine are observed in all brain regions and more NK is quantified (Figure 1B, *n* = 5–6 animals). By 3 h after an IV injection, nearly all ketamine and NK are eliminated from PFC, HPC, and cerebellum (Figure 1C, *n* = 5–6 animals) suggesting the ketamine rapidly reaches brain and is quickly eliminated following injection.

### Ketamine Has No Effect on the Strength of Hippocampal Basal Synaptic Transmission

**Figure 2:**
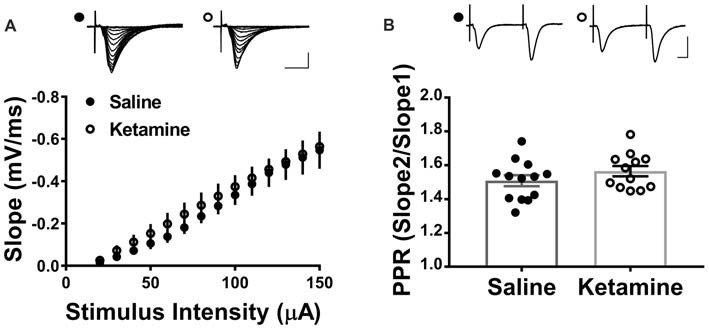
IV ketamine does not alter basal synaptic transmission at CA3-CA1 synapses 3 h post injection. **(A)** No significant difference in stimulus-response curves is observed between ketamine- (*n* = 7 slices/7 animals) and saline-treated rats (*n* = 6 slices/6 animals). **(B)** Paired pulse ratio (PPR) is not significantly different between ketamine- (*n* = 17 slices/12 animals) and saline-treated rats (*n* = 18 slices/13 animals, *p* = 0.22, unpaired *t*-test). Scale bars, 10 ms and 0.5 mV. All values are mean ± SEM.

**Figure 3:**
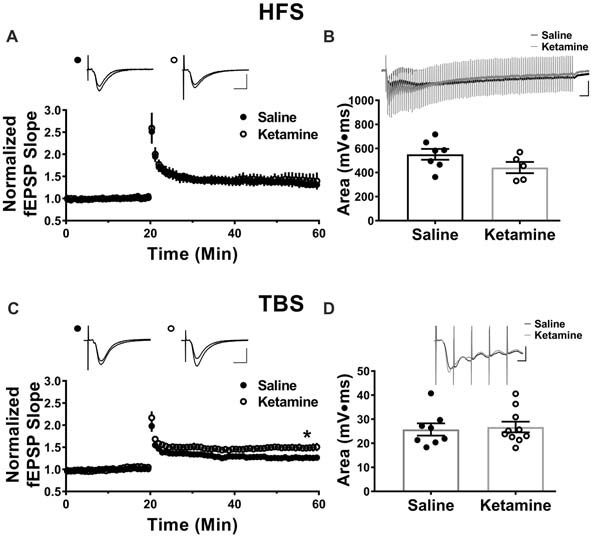
Theta-burst but not high frequency tetanus induced long-term potentiation (LTP) is increased 3 h post IV ketamine treatment. **(A)** No significant difference in LTP magnitude induced using high frequency stimulation (HFS) is observed between ketamine- (*n* = 7 slices/6 animals) and saline-treated rats (*n* = 7 slices/7 animals, *p* = 0.60, unpaired *t*-test). Scale bars, 10 ms and 0.5 mV. **(B)** Ketamine treatment did not significantly change the steady state depolarization during HFS (ketamine-treated *n* = 7 slices/6 animals, saline-treated *n* = 7 slices/7 animals, *p* = 0.98, unpaired *t*-test). Scale bars, 100 ms and 0.5 mV. **(C)** The LTP magnitude induced using theta burst stimulation (TBS) was significantly higher in ketamine- (*n* = 11 slices/10 animals) vs. saline-treated controls (*n* = 11 slices/8 animals, *p* = 0.04, unpaired *t*-test). Scale bars, 10 ms and 0.5 mV. **(D)** No significant difference in steady state depolarization during TBS was observed (ketamine-treated *n* = 11 slices/10 animals, saline-treated *n* = 11 slices/8 animals, *p* = 0.77, unpaired *t*-test). Scale bars, 5 ms and 0.5 mV. All values are mean ± SEM.

The antidepressant effects of ketamine begin within 1–3 h in humans (Berman et al., 2000; Zarate et al., 2006; Fond et al., 2014), and if increased hippocampal function is key to the improved mood, it should be enhanced during this same time frame. To investigate this, we administered ketamine or saline IV in the lateral tail vein of adult male rats and assessed the strength of basal synaptic transmission at CA3-CA1 synapses in stimulus-response curves 3 h post-treatment, using a dose previously shown to have an antidepressant response (Maeng et al., 2008; Li et al., 2010; Autry et al., 2011). No differences in the stimulus-responses curves were observed (Figure 2A ketamine mean = 0.56 ± 0.05 mV/ms, *n* = 7 slices/7 animals, saline mean = 0.55 ± 0.09 mV/ms, *n* = 6 slices/6 animals, *p* = 0.86, unpaired *t*-test at maximum stimulus intensity, 150 μA), indicating that glutamate transmission was not enhanced by ketamine at this early time point. PPR was measured to determine whether ketamine alters presynaptic release probability. During basal transmission, PPR did not differ between ketamine and saline treated rats (Figure 2B ketamine mean = 1.56 ± 0.03, *n* = 17 slices/12 animals, saline mean = 1.50 ± 0.03, *n* = 18 slices/13 animals, *p* = 0.22, unpaired *t*-test) providing indirect evidence that ketamine treatment is not significantly changing release probability. Finally, we found no difference in the magnitude of the steady-state depolarization during the tetanus, with either HFS (Figure 3B ketamine mean = 547.7 ± 113.2, *n* = 7 slices/6 animals, saline mean = 551.2 ± 45.0, *n* = 7 slices/7 animals, *p* = 0.98, unpaired *t*-test) or TBS (Figure 3D ketamine mean = 26.7 ± 2.2, *n* = 11 slices/10 animals, saline mean = 25.7 ± 2.5, *n* = 11 slices/8 animals, *p* = 0.77, unpaired *t*-test) providing additional evidence that basal glutamatergic transmission is not altered 3 h post-IV ketamine.

### LTP Magnitude Is Increased Post-IV Ketamine Only When Induced With TBS

Previous studies reported enhanced LTP magnitude at CA3-CA1 synapses 24 h post-IV ketamine injection (10 mg/kg and 3 mg/kg; Burgdorf et al., 2013; Graef et al., 2015). Here, we examined whether enhanced LTP is observed as soon as 3 h post-IV ketamine, a time point that correlates with the earliest improvement in depressive symptoms (Berman et al., 2000; Zarate et al., 2006; Fond et al., 2014). Additionally, we used both HFS and TBS to induce LTP, as these different stimulation patterns activate different intracellular pathways to cause long lasting potentiation (Zhu et al., 2015). Three hours post-IV injection, the LTP magnitude induced using HFS (two 100 Hz trains at 1 s duration, 20 s interval between trains) did not differ between ketamine- and saline-treated male rats (Figure 3A ketamine mean: 1.41 ± 0.17, *n* = 7 slices/6 animals, saline mean: 1.32 ± 0.06, *n* = 7 slices/7 animals, *p* = 0.60, unpaired *t*-test). In contrast, the LTP magnitude induced using TBS (four trains of 10 bursts consisting of five pulses at 100 Hz, 200 ms intraburst interval, 20 s interval between trains) was significantly higher in ketamine vs. saline-treated controls (Figure 3C ketamine mean: 1.49 ± 0.08, *n* = 11 slices/10 animals, saline mean: 1.26 ± 0.04, *n* = 11 slices/8 animals, *p* = 0.04, unpaired *t*-test). Comparison of the magnitude of LTP in the HFS and TBS saline-treated rats yielded no significant difference (HFS induced LTP: 1.32 ± 0.06, *n* = 7 slices/7 animals, TBS induced LTP: 1.26 ± 0.04, *n* = 11 slices/8 animals, *p* = 0.47, unpaired *t*-test), showing that the type of stimulation used did not affect the overall magnitude of LTP.

### Ketamine Has No Effect on Spine Density in Hippocampus or Prefrontal Cortex 3 h Post-IV Injection

**Figure 4:**
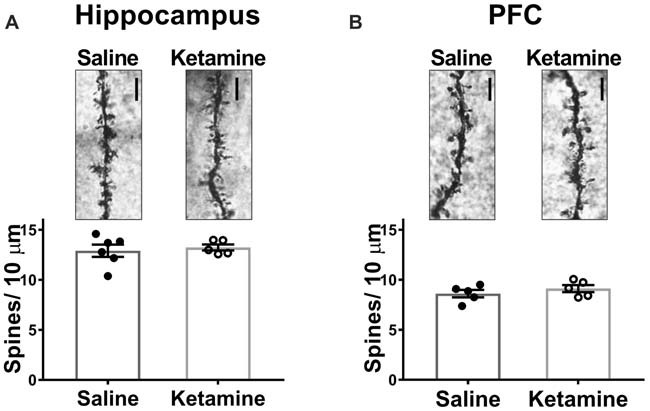
Spine density in HPC or PFC is not altered 3 h post IV ketamine injection. No significance difference in spine density is measured between ketamine- and saline-treated rats in HPC **(A)** (ketamine-treated *n* = 5 animals/35 dendrites, saline-treated *n* = 6 animals/40 dendrites, *p* = 0.66, unpaired *t*-test) or PFC **(B)** (ketamine-treated *n* = 5 animals/35 dendrites, saline-treated *n* = 5 animals/35 dendrites, *p* = 0.35, unpaired *t* test). Scale bars, 5 μm. All values are mean ± SEM.

Decreased dendritic spine density is implicated in the pathophysiology of MDD, and many studies have examined whether antidepressant treatments can reverse this (Duman, 2014), with most studies examining morphological effects of ketamine 24 h post-injection. In PFC, spine density of layer V pyramidal cells is increased (Li et al., 2010), and spine density was also increased in area CA1 with ketamine treatment (Liu et al., 2016). However, investigation of spine density changes immediately after ketamine treatment is limited. One recent study demonstrated that ketamine increased spine density in PFC 3 h post treatment (Sarkar and Kabbaj, 2016). We examined spine density changes in area CA1 of HPC and in medial PFC 3 h post-IV injection. No significant difference was detected between ketamine- and saline-treated rats in either brain region (Figure 4A ketamine mean: 13.24 ± 0.31 spines/10 μm, *n* = 5 animals/35 dendrites, saline mean: 12.91 ± 0.61 spines/10 μm, *n* = 6 animals/40 dendrites, *p* = 0.66, unpaired *t*-test; (Figure 4B ketamine mean: 8.62 ± 0.37 spines/10 μm, *n* = 5 animals/35 dendrites, saline mean: 9.13 ± 0.35 spines/10 μm, *n* = 5 animals/35 dendrites, *p* = 0.35, unpaired *t*-test). These data imply that connectivity in the form of increased synapse density is not altered at this early time point after ketamine treatment.

## Discussion

Here, we report IV ketamine treatment enhances TBS induced LTP without changing the strength of basal synaptic transmission or HFS induced LTP at CA3-CA1 synapses in adult male rats. While *in vitro* studies show synaptic transmission is enhanced within 1 h of bath application of ketamine (20 μM; Autry et al., 2011; Nosyreva et al., 2013; Izumi and Zorumski, 2014), we observed no difference in synaptic transmission 3 h after IV treatment. This discrepancy may be due to differences in *in vivo* and *in vitro* ketamine concentration, as the IV injection likely does not reach 20 μM. Although there was no difference in basal transmission, increased synaptic transmission via activity-dependent plasticity following ketamine treatment suggests ketamine is priming hippocampal circuits and increasing the response of CA1 pyramidal cells to synaptic activity.

Interestingly, enhanced plasticity was only observed in slices from ketamine-treated animals when TBS was used as the LTP-inducing stimulus. Previous studies demonstrate that TBS and HFS activate different intracellular pathways (Zhu et al., 2015), and that TBS LTP is more reliant on BDNF signaling than HFS induced LTP (Chen et al., 1999; Patterson et al., 2001; Edelmann et al., 2015; Aarse et al., 2016). This is an important distinction because ketamine has been shown to increase activity-dependent release of BDNF, and the antidepressant-like effects of ketamine rely on BDNF (Lepack et al., 2014). Therefore, ketamine is potentially increasing BDNF levels in HPC that underlie the enhanced TBS LTP magnitude compared to saline-treated animals. However, we cannot exclude that other intracellular pathways may be involved, and future studies are warranted to assess whether BDNF is involved in the enhanced TBS LTP.

The lack of increase in spine density is consistent with the lack of increase in the strength of basal transmission measured in the stimulus-response curve. Additionally, the morphological changes induced by ketamine treatment may rely on increased function, such as increased LTP, and appear at later timepoints. However, a previous study reported ketamine treatment increased spine density in PFC 3 h post injection (Sarkar and Kabbaj, 2016). The disagreement between our finding and this previous study could be due to different methods of labeling. We are not able to select which cells are stained using the Golgi method, and it is possible only a small subset of cells had increased spine density, which would be lost in the overall population.

A previous study suggests the antidepressant effect of ketamine is through a metabolite, hydroxynorketamine (HNK; Zanos et al., 2016). Despite this, it remains unclear whether the brain HNK concentration would reach the effective concentration following ketamine IV infusion in patients (Collingridge et al., 2017). Here, we report ketamine is eliminated from brain within 3 h and levels of the metabolite, NK, reach a fraction of the ketamine concentration in brain, supporting the concept of ketamine having an initial trigger in brain that leads to lasting molecular and structural changes. Ketamine likely causes a transient increase in glutamatergic transmission as *in vitro* studies indicate ketamine increased CA3-CA1 synaptic responses (Nosyreva et al., 2013; Izumi and Zorumski, 2014; Zhang et al., 2016) and disinhibited CA1 pyramidal cells (Widman and McMahon, 2018). This may lead to release of more BDNF, which allows for the enhanced LTP we observe at CA3-CA1 synapses. Additionally, LTP can lead to increase protein synthesis, which is observed hours after ketamine treatment (Maeng et al., 2008; Li et al., 2010, 2011; Autry et al., 2011; Burgdorf et al., 2013; Nosyreva et al., 2013). Together, this series of functional and cellular changes could lead to the antidepressant behavioral effects that last for days.

## Author Contributions

AW, EE, EG and LM designed research. AW, AS and EE performed research. AW, AS, EE, EG and LM analyzed data. AW and LM wrote the article.

## Conflict of Interest Statement

The authors declare that the research was conducted in the absence of any commercial or financial relationships that could be construed as a potential conflict of interest.
